# Associations between adverse childhood experiences and history of weight cycling

**DOI:** 10.1002/osp4.736

**Published:** 2024-02-16

**Authors:** Caitlin E. Smith, Kelsey L. Sinclair, Temitope Erinosho, Andrew C. Pickett, Vanessa M. Martinez Kercher, Lucia Ciciolla, Misty A. W. Hawkins

**Affiliations:** ^1^ Department of Psychiatry Yale University New Haven Connecticut USA; ^2^ Department of Health & Wellness Design Indiana University Bloomington Indiana USA; ^3^ Department of Applied Health Science Indiana University Bloomington Indiana USA; ^4^ Department of Psychology Oklahoma State University Stillwater Oklahoma USA

**Keywords:** adverse childhood experiences, early life adversity, obesity, overweight, weight cycling

## Abstract

**Background:**

Adverse childhood experiences (ACEs) predict obesity onset; however, the relationship between ACEs and history of weight cycling has not been adequately explored. This gap is problematic given the difficulty in weight loss maintenance and the impact of ACEs on obesity development, chronicity, and associated weight stigma. The objective of this study was to examine associations between self‐reported history of ACEs and weight cycling in a sample of weight loss treatment‐seeking adults with overweight/obesity.

**Methods:**

The number of participants in the analyzed sample was 78, mostly white educated adult women (80% female, 81% Caucasian, 75% ≥ bachelor's degree) with excess adiposity enrolled in the Cognitive and Self‐regulatory Mechanisms of Obesity Study. ACEs were measured at baseline using the ACEs Scale. History of weight cycling was measured using the Weight and Lifestyle Inventory that documented weight loss(es) of 10 or more pounds.

**Results:**

Higher ACE scores were associated with a greater likelihood of reporting a history of weight cycling. Participants with four or more ACEs had 8 times higher odds (OR = 8.301, 95% CI = 2.271–54.209, *p* = 0.027) of reporting weight cycling compared with participants with no ACEs. The association of weight cycling for those who endorsed one to three ACEs was not significant (OR = 2.3, 95% CI = 0.771–6.857, *p =* 0.135) in this sample.

**Conclusions:**

The role of ACEs in health may be related to associations with weight cycling. Results indicated that those who reported four or more ACEs had significantly higher odds of reporting weight cycling compared with those with no ACEs. Further research is needed to further explore how ACEs predict the likelihood of weight cycling, which may be prognostic for sustained weight loss treatment response and weight stigma impacts.

## Graphical abstract

Adverse childhood experiences (ACEs) were linked to weight cycling among a treatment‐seeking sample of overweight/obesity. Specifically, participants endorsing four or more ACEs were 8 times more likely to report weight cycling.

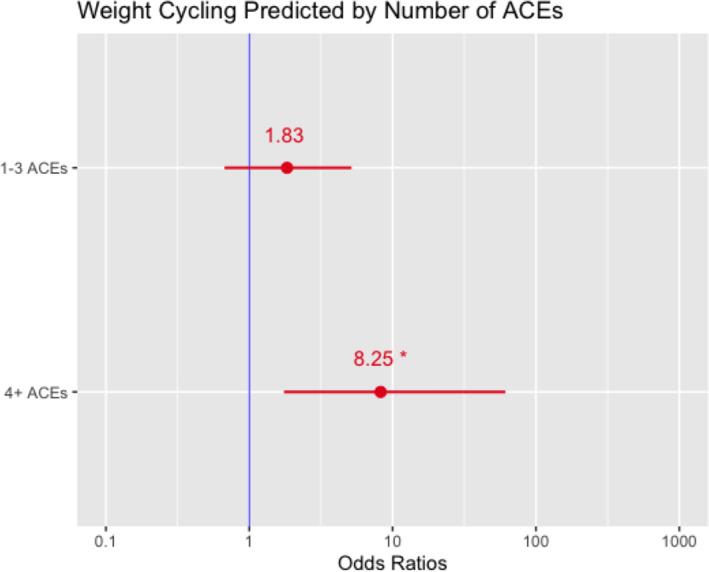

## 1 INTRODUCTION

Adverse childhood experiences (ACEs) and overweight/obesity are two highly prevalent and interrelated predictors of adverse biopsychosocial health effects.^1^, ^2^, ^3^, ^4^ ACEs are defined as traumatic events or maltreatment experienced prior to 18 years of age, such as deprivation/neglect, household challenges, and/or abuse,^4^ whereas obesity is defined using body mass index (BMI) of 30 kg/m^2^ or greater.^3^ The ACE Study Survey^4^ suggests that 64% of participants have experienced at least one ACE prior to the age of 18 years, while 72% of adults aged 20 or older qualify as overweight or obese.^3^

Both ACEs and obesity have shown dose‐response effects on health, such that as a person's ACE score or BMI increases, multiple indices of their health worsen. Threshold effects are also detected, such that ≥4 ACEs and BMIs ≥30 typically confer clinically meaningful adverse health effects.^5^, ^6^, ^7^, ^8^ ACEs have been linked to obesity since the original ACE study^4^ that explored a history of ACEs as a potential reason for the high dropout rates at an obesity treatment clinic.^9^ Since these origins, a growing evidence base shows that ACEs and obesity are cross‐sectionally and epidemiologically related, such that regions in the United States with the highest obesity rates also tend to have the highest rates of ACEs.^10^, ^11^ Prospectively, ACEs also predict obesity onset up to 30 years later,^12^ with estimates that individuals with ACEs are 1.4 times more likely to develop obesity.^13^ Several mechanistic pathways can explain the ACEs‐obesity relationship. Exposure to childhood poverty and food insecurity have been associated with ACEs and are predictors of adulthood obesity. ACEs have also been linked to impairments in self‐regulation, which can lead to obesogenic eating behaviors.^14^, ^15^, ^16^ ACEs are also related to increased risk of internalized weight stigma and body‐related shame, which are in turn related to physical activity avoidance behaviors.^17^, ^18^, ^19^, ^20^, ^21^, ^22^ Despite evidence of an ACEs‐obesity relationship and plausible mechanisms, few investigations have examined whether ACEs are related to a history of weight cycling in samples of individuals with overweight or obesity.

Weight cycling is broadly defined as repeated weight losses followed by weight regain—though the number and magnitude of these loss/regain cycles vary across studies. Worldwide, an estimated 44% of the general population has reported a weight loss attempt,^23^ and the evidence suggests that the majority of these individuals will experience weight regain—making weight cycling a highly prevalent phenomenon.^23^, ^24^ Indeed, 20%–35% of men and 20%–55% of women endorse weight cycling^24^, with adults endorsing progressively more frequent weight loss attempts than in prior years.^25^ Weight cycling has a reputation for adverse physiological impacts (e.g., metabolic slowing or complications) with some empirical support.^26^, ^27^ However, systematic reviews have also suggested that weight cycling does not have a substantial negative metabolic impact, especially among persons with overweight or obesity.^28^ Research in animal models suggests that weight‐cycling may even be beneficial for longevity, as a clever study of weight‐cycling mice showed that they outlived their rodent peers with sustained weight loss.^29^

Although animal models can inform the physiological effects of weight cycling to some degree, one of their primary criticisms is that the biopsychosocial impacts of weight cycling are not fully captured within current models. Specifically, rodents and other non‐human species do not exhibit analogous weight stigmatizing behaviors to those that their human counterparts display. For instance, human children as young as three will describe overweight children as “stupid,” “lazy,” or “ugly”^30^ while one might assume that rat pups are not negatively labeling their overweight peers. Likewise, adult mice do not write newspaper articles asserting that fat people should be confined to “prison camps,” both for their own and societal good.^31^ While these two examples are somewhat tongue‐in‐cheek, they highlight that—when possible—weight cycling research should account for the stressful interpersonal context and reality of weight stigma or discrimination (a type of sizeism) to be more applicable to humans.

Weight stigma (both internalized and experienced) is the social devaluation of a person based on their body weight.^32^ Weight stigma impacts individuals at both ends of the weight spectrum and is an established stressor with biochemical effects that are independent of adiposity levels, including blunted cortisol reactivity and greater oxidative stress.^32^, ^33^, ^34^, ^35^, ^36^ Weight stigma may partially motivate weight loss attempts and make weight regain feel threatening, as people become more vigilant to avoid weight‐based devaluation.^37^ Indeed, greater weight cycling is related to greater depressive symptoms, with internalized weight stigma as a partial mediator of this association,^38^ which has relevance and provides a rationale for the present study given the connection between higher ACEs and greater internalized weight stigma.^21^

In addition to depression, weight cycling is linked to anxiety and body image dissatisfaction as well as levels of cortisol, oxidative stress, and C‐reactive protein^39^, ^40^—all linked to chronic stress activation and diminished mental health. Given the role of chronic stress in heightened mortality risk,^41^ research examining weight cycling should account for its biopsychosocial effects. Thus, despite the controversy over its biological harms within certain domains (e.g., metabolism) and its longevity‐enhancing effects in rodent models, weight cycling has been associated with psychophysiological harms,^39^, ^40^ higher current and historical BMIs with risk of sarcopenic obesity,^42^ greater reward sensitivity,^43^ and is potentially problematic for those seeking sustained weight loss^44^ or avoidance of the harms of weight‐based devaluation.^37^

The goal of this study is not to answer in which context weight cycling may be harmful but to better understand ACEs as a potential predictor of this phenomenon. Knowing whether a history of ACEs is associated with a history of weight cycling may inform which groups are at greater risk for weight regain cycles. Understanding these risks may ultimately point to modifiable targets to promote more sustainable weight loss for those who seek it or greater body acceptance or self‐esteem for those who don't. For example, some authors have suggested that a history of weight loss attempts does not cause more weight gain than that would have occurred in the absence of the attempts.^44^ Knowing whether ACEs drive some of this propensity for gaining excess weight that is treatment‐resistant would be useful. Previous work suggested that higher ACEs were linked to higher BMIs and greater abdominal adiposity in a treatment‐seeking sample of adults with overweight/obesity.^45^ The present study expands these results by examining the associations between a history of self‐reported ACEs and weight cycling. It is hypothesized that persons with higher ACE scores would be more likely to endorse a history of weight cycling.

## 2 METHODS

*Overview*. The Cognitive and Self‐regulatory Mechanisms of Obesity Study (COSMOS) trial (NCT02786238) was a multi‐year, multi‐cohort pilot trial with 108 enrolled participants (aged 21–65 years old). The parent project was a comparative effects pilot examining how two behavioral weight loss interventions impact physical, neurocognitive, and self‐regulation factors. The data and analyses from the current project are limited to the baseline data from this trial and included 78 participants with ACE scores and weight cycling survey data. The 30 participants with missing data did not turn in the packet with the ACEs and weight cycling surveys so were not included in the analyses. Full details about the parent study methodology can be found in the published trial protocol paper.^46^

*Adverse Childhood Experiences*. ACEs were measured using the ACEs Survey.^47^ The ACEs Survey asks participants to indicate whether they have experienced 10 possible traumatic events occurring before age 18, including emotional, physical, and sexual abuse, emotional and physical neglect, domestic violence, parental separation/divorce, familial mental illness, substance use, and/or incarceration. In addition to using a total ACE score, individuals were characterized into one of three groups based on their ACE score (0, 1–3, or 4+ ACEs). Examining the potential threshold effect of 4+ ACEs compared with fewer (1–3) and no ACEs (0) is consistent with previous research examining threshold effects.^48^, ^49^

*Weight Cycling*. To assess weight cycling participants completed the Weight and Lifestyle Inventory (WALI).^50^ The WALI is designed to gather information about weight and dieting habits, eating and exercise habits, and relationships with family friends. To assess weight cycling specifically, section E of the WALI was utilized. In this section, participants record major weight loss efforts that resulted in a weight loss of 10 or more pounds as well as record the age during the effort and methods used to lose weight. The number of efforts was summed by the researchers. It is important to note that this section of the inventory has not been validated to measure weight cycling behavior.

*Adiposity Variables and Covariates*. Participants completed baseline self‐report questionnaires assessing relevant covariates, including age (years), gender (0 = male, 1 = female), race‐ethnicity (white, Black or African American, American Indian or Alaskan Native, Asian/Pacific Islander, Hispanic/Latino), and education level (middle school, high school, some college, associate's, bachelor's, graduate or professional). BMI (kg/m^2^) was obtained using measured height and weight from a standard medical scale to the nearest 0.1 of the kg. Participants were weighed wearing casual clothes and without shoes. Waist circumference was measured in centimeters according to the World Health Organization guidelines.^51^ Height, weight, and waist circumference were measured in the lab by study staff.

*Procedure*. Participants were recruited from the local university/community and completed online/phone screenings before the COVID‐19 pandemic. Eligible participants who wanted to enroll provided written informed consent and were scheduled for their baseline assessments, which included a series of self‐report questionnaires (including the ACEs Survey) and a laboratory testing session administered by trained research staff. Participants received $75 reimbursement for completing the visit.

*Statistical Methods*. All statistical tests were run using IBM SPSS Statistics software. The total ACE score was examined, and individuals were also categorized into one of three groups based on their ACE score (0, 1–3, or 4+ ACEs). Binomial and logistic regression tests were used to examine the relationship between ACE total score and ACE group differences across individuals who reported weight cycling. Age, education level, and baseline BMI were used as covariates. Participants reporting no ACEs were used as the reference group in the regressions.

## 3 RESULTS

Of the 108 enrolled participants, 30 failed to provide valid data regarding weight cycling. Of the remaining sample (*n* = 78), 43 reported a history of weight cycling (55%) and 35 reported no history of weight cycling (45%). Of the ACE groups, 32% of participants reported having no ACEs (*n* = 25), 51% reported 1–3 ACEs (*n =* 40), and 17% reported 4 or more ACEs (*n* = 13). See Table 1 for participant characteristics.

**TABLE 1:** Characteristics of participants.

	Total analyzed sample *(n =* 78)	No ACEs (*n* = 25)	1–3 ACEs (*n* = 40)	4+ ACES (*n* = 13)
Demographic factors				
Age (years)	45.59 ± 11.52	46.12 ± 10.49	43.60 ± 11.07	44.69 ± 15.00
Sex (% female)	79.5	84.0	77.5	76.9
Race/Ethnicity (% white)	80.8	80.0	80.0	84.6
Education (% bachelors)	34.6	36.0	35.0	30.8
Weight cycled (% yes)	55.1	40.0	55.0	84.6
Number of weight cycles	1.01 ± 1.27	0.68 ± 1.03	1.08 ± 1.44	1.46 ± 1.05

**FIGURE 1:**
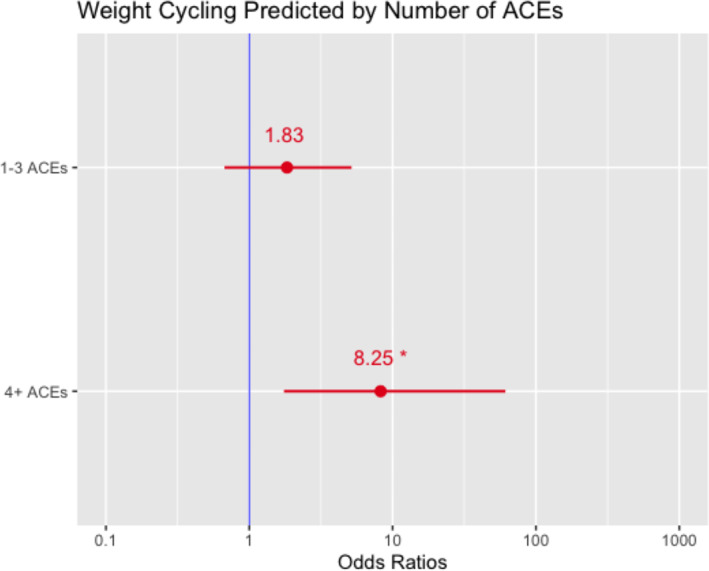
Odds of Reporting Weight Cycling Across Groups Endorsing a History of ACES. Referent group: individuals with zero ACES. ACES, adverse childhood experiences.

A binomial logistic regression revealed a significant relationship between ACE total score and weight cycling behavior (OR = 1.475, 95% CI = 1.038–2.095, *p* = 0.03). Baseline BMI was also significantly related to weight cycling (OR = 1.113, 95% CI = 1.015–1.220, *p* = 0.022). When this relationship was trisected using ACE categories, results indicated that—compared to participants who had no ACEs, individuals with 4+ ACEs had 8 times higher odds of reporting weight cycling compared to those with no ACEs (OR = 8.301, 95% CI = 2.271–54.209, *p* = 0.027) (see Figure 1). In this pilot sample, there was no significant association in weight cycling for participants with 1–3 ACEs (OR = 2.300, 95% CI = 0.771–6.857, *p =* 0.135) (see Figure 1).

## 4 DISCUSSION

Results indicated that a history of ACEs predicted an increased likelihood of weight cycling, especially for individuals who had accumulated four or more ACEs. Specifically, those with four or more ACEs had 8.3 times higher odds of reporting weight cycling than those who had not experienced any ACEs. There was a trend toward a potential dose‐response‐type relationship, such that those who endorsed 1 to 3 ACEs may also be more likely to report a history of weight cycling than those with no ACE history (twice as likely), but this relationship was not statistically significant. The failure to detect an association in this group may be due to insufficient power in this sample. Informed by the observed effects in the present study (OR = 2.3), a sample of 78 individuals with 1–3 ACEs would be expected to detect an effect at 80% power (with an alpha of 0.05, covariates accounting for 12% of the effect, and base rate of target event at 0.30). Given that the 1–3 ACEs group was only 40 individuals in this study, future studies with larger samples and greater diversity in ACE history can determine whether lower ACE thresholds (i.e., <4) are still associated with greater weight cycling.

Direct prospective investigations of ACEs and weight cycling have not been conducted. However, the literature supports longitudinal relationships between ACEs and future obesity. A review found that exposure to multiple ACEs increased the odds of developing obesity in adulthood by 46%.^52^ As weight loss continues to be the recommendation for the treatment of obesity, it is estimated that up to 40% of Americans are attempting to lose weight and approximately 20% of men and 40% of women have completed at least one instance of weight cycling.^23^ Given the close interrelationship of ACEs and obesity, the current findings support that weight cycling may also be an ACE‐related health pattern. Such patterns may impact weight loss success, even in the context of surgical weight loss. Investigations of ACEs and surgical weight loss interventions have shown mixed results. Some studies do not show significant differences in weight loss between those with ACEs and those without.^53^, ^54^ However, others have indicated that those with a high number of ACEs (>6) experienced worse outcomes compared to those with fewer ACEs.^55^ A meta‐analysis reported that behavioral weight loss interventions tailored for individuals with a history of ACEs are not available despite the potential impact of ACEs on sustainable weight loss.^56^ Therefore, literature on the impact of ACEs on surgical and behavioral weight loss treatment response remains underdeveloped.

Based on these results, those who experience more ACEs have a higher odds of reporting weight cycling. ACEs are related to multiple negative health behaviors in adulthood (e.g., poor nutrition, increased sedentary behavior, reduced physical activity) that are independently associated with and/or exacerbated by obesity when combined.^57^, ^58^, ^59^ However, in addition to ACEs‐related associations with these behaviors, the ACEs‐weight cycling relationship may be partially due to stress‐based mechanisms. One study found that experiencing ACEs has been linked to adult psychological stress. Experiencing 1–5 ACEs increases the odds of developing psychological stress by three times compared with those with no ACEs. When increased to six or more ACEs the odds were eight times more likely of developing psychological distress.^60^ This relationship was further impacted by life stressors. Management of stress and coping along with psychological stability have been associated as factors related to weight loss maintenance.^61^ Weight maintenance is difficult to achieve, with approximately 95% of people who have lost weight relapsing and regaining within a year.^25^ Those with more ACEs may have further difficulty in maintaining weight and may be at risk to the weight cycle due to this process, as higher ACEs have been associated with greater distress. Research is needed to further examine the underlying relationship.

Associations between weight cycling and ACEs have not been fully explored. This study provides the impetus for future research on the strong associations between the likelihood of weight cycling and history of ACEs and on the potential mechanisms of these associations. Notably, this study had key limitations. First, this study utilized a small sample comprised of highly educated white females. Therefore, findings may not be generalizable to the general population and additional research is needed to fully understand the implications of ACEs on weight cycling for males and across various socio‐economic status levels and/or other marginalized groups. Additionally, the sample of individuals reporting four or more ACEs was small, and the pattern of results may differ when examining weight cycling within larger groups of individuals with high ACEs. Also, the measure used to assess weight cycling was not a validated measure, so reporting and results may not be as accurate. Future directions should also examine the role of ACEs in behavioral weight loss interventions, as their impact is underexplored, and the results indicate that ACEs may play a role in weight loss maintenance. Such work might be implemented by 1) consistently adding ACE screenings to behavioral weight loss programs as a priori treatment heterogeneity factors to be explored, and 2) adapting or incorporating trauma‐informed care strategies such as Mollard and Hudson's four E model^62^ to weight loss interventions to determine their impact on weight loss success among those with different ACE levels. With regards to animal models, perhaps creative iterations can be tested in which existing social defeat/chronic stress paradigms^63^, ^64^ are integrated with experimentally manipulated eating regimens. For example, eating behaviors or weight gain could be consistently linked to social stress stimuli across the lifespan in order to more closely approximate the human experience of being exposed to early life stress, the devaluation of the one's worth based on having a larger body size, and then losing and regaining weight in these interpersonal contexts.

## 5 CONCLUSION

This exploratory study suggests that ACEs may play a role in weight cycling behavior. Specifically, experiencing more ACEs (especially four or more) significantly predicted higher odds of weight cycling. Previous literature has connected ACEs to the development of obesity but has not determined the impact of ACEs on sustainable weight loss. Further research is needed to understand how the history of ACEs and its treatment can impact weight loss and maintenance or other weight‐related experiences, such as weight stigma.

## CONFLICT OF INTEREST STATEMENT

The authors declare no conflicts of interest.
